# Enhanced recovery after surgery protocol optimizes results and cost of laparoscopic radical nephroureterectomy

**DOI:** 10.1186/s12893-025-02758-3

**Published:** 2025-01-09

**Authors:** Jiageng Shi, Siming Chen, Jiawei Nie, Kangping Xiong, Gang Wang, Kaiyu Qian, Hang Zheng, Xinghuan Wang

**Affiliations:** 1 Department of Urology, Zhongnan Hospital of Wuhan University, Wuhan, China; 2 Department of Biological Repositories, Zhongnan Hospital of Wuhan University, Wuhan, China; 3 Laboratory of Precision Medicine, Zhongnan Hospital of Wuhan University, Wuhan, China; 4 Human Genetics Resource Preservation Center of Hubei Province, Wuhan, China; 5 Medical Research Institute, Wuhan University, Wuhan, China

**Keywords:** Enhanced recovery after surgery (ERAS), Upper tract urothelial carcinoma (UTUC), Laparoscopic radical nephroureterectomy (LRNU), Length of hospital stay (LOS), Postoperative complications

## Abstract

**Purpose:**

To evaluate the efficacy of an enhanced recovery after surgery (ERAS) strategy for upper tract urothelial carcinoma (UTUC) patients undergoing laparoscopic radical nephroureterectomy (LRNU).

**Methods:**

90 patients who received LRNU at Zhongnan Hospital of Wuhan University between January 2018 and July 2022 were retrospectively analyzed, including 43 in the ERAS group and 47 in the pre-ERAS group. The clinical features, postoperative complications, length of hospital stay (LOS), and hospital expenditures of the two groups were compared via t-test, Mann-Whitney test, and Chi-square test.

**Results:**

In comparison to the pre-ERAS group, the total and postoperative LOS were significantly shorter in the ERAS group [total LOS: 15.0 (13.0–20.0) vs. 21.0 (16.0–26.0), *p* < 0.001; postoperative LOS: 8.0 (7.0–9.0) vs. 11.0 (9.0–13.0), *p* < 0.001]. The ERAS group had lower hospitalization costs than that in the pre-ERAS group [56896.40 (48324.30-67498.01) vs. 64249.83 (55574.36-81581.82), *p* = 0.010]. Additionally, the ERAS group experienced a reduction in postoperative fever incidence (23.4% vs. 4.7%, *p* = 0.011).

**Conclusions:**

In the realm of LRNU, ERAS protocols are safe and practical for minimizing the LOS while accelerating the rehabilitation of patients undergoing LRNU. This study offers insights for enhancing ERAS protocols for UTUC patients even further.

**Supplementary Information:**

The online version contains supplementary material available at 10.1186/s12893-025-02758-3.

## Introduction

Upper tract urothelial carcinoma (UTUC) is a relatively rare type of urological cancer that comprises renal pelvic carcinoma and ureteral carcinoma and accounts for 5–10% of all urothelial cancers [1]. The standard surgery for high-risk non-metastatic UTUC is radical nephroureterectomy (RNU) with removal of bladder cuff excision [2]. Since the 1991, when Clayman et al. originally detailed the first laparoscopic radical nephroureterectomy (LRNU), LRNU has become a popular surgical intervention with reduced postoperative discomfort and recovery time [3, 4]. The surgical procedure of LRNU may be performed with two different approaches: peritoneal and retroperitoneal approaches, and in this study, we chose patients with UTUC who received LRNU by retroperitoneal approach. The retroperitoneal route allows more direct and rapid access to the corresponding organs and lesions, which is more advantageous for the exposure and treatment of renal arteries and veins, while avoiding damage to the large abdominal vessels and organs, but the small space for retroperitoneal operation makes the treatment of the lower ureter difficult [5]. For the management of the end of the ureter, we used a combined lower abdominal oblique incision open surgery [6].

Enhanced recovery after surgery (ERAS) refers to the notion of using a series of optimized measures proven to be effective in the perioperative period to accelerate the patient’s postoperative recovery [7]. ERAS has developed rapidly in recent years and is widely recognized in neurosurgery, colorectal surgery, hepatobiliary surgery, urology and other specialties to reduce hospitalization time, medical expenses and complications after surgery [8–11]. While ERAS has been shown to yield good results in other urological procedures, the data for LRNU is scant [12–14].

In order to aid in future clinical decision-making, we undertook a retrospective analysis to compare the postoperative length of stay, hospital expenditures, and postoperative complications of UTUC patients undergoing LRNU prior to and following deployment of ERAS.

## Methods

### Patients

We retrospectively assessed UTUC patients who received LRNU at our center from January 2018 to July 2022. The ERAS protocol was implemented from September 1, 2020, after which all patients receiving LRNU were on the ERAS protocol. All patients had no preoperative distant metastases, were treated with LRNU, and had a postoperative pathological diagnosis of UTUC. The exclusion criteria: (1) Postoperative pathology suggesting non-uroepithelial carcinoma; (2) Combination of additional malignancies; (3) Severe cardiovascular disease or pulmonary dysfunction; (4) No complete clinical data. 90 patients were included in the study; 47 were in the pre-ERAS group and 43 were in the ERAS group.

### Surgical technique

All patients underwent retroperitoneal LRNU combined with lower abdominal oblique incision and sleeve cystectomy and all surgeries were performed by high-level surgeons within our department’s oncology treatment group. Combined inhalation and intravenous anesthetics were used to treat all patients, and a ventilator was used to maintain ventilation. After general anesthesia, a 10 mm trocar was positioned in the mid-axillary line, 2 cm above the iliac crest (to place the laparoscope), a 12 mm trocar was positioned in the same location in the anterior axillary line, and a 5 mm trocar was positioned in the posterior axillary line, under the twelfth rib border. The kidney and the middle and upper ureter were laparoscopically freed via retroperitoneum in the lateral position following that, a retroperitoneal drainage tube was inserted. A Gibson cut was then performed in the lower abdomen on the affected side of the patient while they were lying on their back. The ureter on the affected side was freed upward, and the kidney, ureter and part of the bladder were completely removed and a drainage tube was inserted in the pelvis.

### Pre-ERAS management

All patients received traditional perioperative management. Patients underwent a 12-hour fast and a 4-hour water restriction and a cleansing enema prior to the procedure. Nasogastric intubation was not used during the surgical procedure. Patients received free fluid therapy during the operation, and postoperatively, patients started drinking after anal exhausted and gradually transitioned to a normal diet. Patients rested in bed routinely on the first day after surgery, and then started bed activities until walking according to the patients’ wishes.

### ERAS management

A reasonable care plan was developed on the basis of the state of the patient before surgery. Preoperatively, doctors and nurses informed patients and their families of the meaning and content of ERAS, instructed them on relevant precautions, communicated fully with them, and relieved patients’ anxiety and bad mood. Patients fasted for 6 h before surgery and took 300 ml of dross-free carbohydrate orally 2 h before surgery. Preoperative cleansing enemas were not required. Nasogastric intubation was not used during the surgical procedure. During the operation, the body temperature was closely monitored, the infusion temperature was controlled, and the body surface heating blanket device was used. Increase the use of local infiltration anesthesia for the incision during surgery, and use NSAIDs for analgesia on time after surgery, avoiding opioid application as possible. After recovering from surgical anesthesia, patients can sip water, and take liquid food on the first postoperative day and gradually transition to a normal diet. Intermittent pneumatic compression device was used to prevent venous thrombosis of lower limbs after surgery. The key distinctions between pre-ERAS management and ERAS management are outlined in Table 1.

**Table 1:** Enhanced recovery protocol for laparoscopic radical nephroureterectomy

Managements	pre-ERAS	ERAS
Education	Routine preoperative education	More comprehensive preoperative education, including a rapid recovery program that guides patients to early eating and early activity, etc.
Nurse	Primarily concerned with postoperative care operations	Focus on the entire perioperative care, including preoperative education and postoperative psychological and medical care
Preoperative fasting (h)	12	6
Preoperative water deprivation (h)	4	2 (drink 300 ml of dross-free carbohydrate orally 2 h before surgery)
Bowel preparation	Cleansing enema the night before and the morning of the operation	No enemas
Postoperative fasting (h)	Until bowel movements resume	6 after surgery
Postoperative water deprivation	POD1	Awakening from anesthesia
Intraoperative intravenous fluid intake	General about 1500 ml	Communicate with anesthesiologist to minimize fluid input
Postoperative intravenous fluid intake	Complete intravenous fluids on POD1;	Generally no more than 1000 ml on POD1;
Intraoperative warming	Not emphasized	Preheat intravenous transfusion fluids or warm blankets
Postoperative analgesia	Postoperative on-demand analgesia	Local anesthetic incision infiltration is used. Timely postoperative analgesia with NSAIDs, avoiding opioids as much as possible
Postoperative activities	Rested in bed on the POD1. Usually walk on the POD2 or POD3	Sit and walk on the POD1

POD, postoperative day

### Data collection

The basic data, perioperative data and pathological data of the patients were collected (Supplementary Material 1). The drainage tube removal time refers to the removal time of both drainage tubes. In this study, the LOS included both the total hospital stay and the postoperative hospital stay. The total LOS and postoperative LOS were computed from admission and postoperative to discharge for both LOS categories. Hospitalization costs are all medical expenses incurred during a patient’s hospitalization, and can be divided into direct medical expenses and indirect medical expenses. Direct medical expenses include items directly related to treatment, such as medical service fees (diagnosis and treatment), drug costs, examination and laboratory fees (including various clinical examinations, laboratory tests, and imaging tests), and surgical costs (surgery, operation, anesthesia, etc.). Indirect medical expenses include items indirectly related to treatment but related to the patient’s recovery, such as nursing costs, hospital bed costs (based on the number of days of hospitalization), and consumables (disposable medical supplies, surgical instruments, etc.). Early postoperative complications include delirium, urinary retention, fever, gastrointestinal distress, postoperative hypotension, and postoperative cardiovascular accidents. All patients were not rehospitalized for postoperative complications within 30 days of discharge.

### Statistical analysis

SPSS 20 was used for the statistical analysis. The Shapiro-Wilk test was employed to validate the normality of the continuous variable distribution. All these normally and nonnormally distributed data were evaluated separately via Student’s t-test and the Mann-Whitney test. Mean values and standard deviations were used to represent continuous data with normal distributions, whereas medians and interquartile ranges were used to report continuous variables with nonnormal distributions. The categorical variables were compared via Fisher’s exact test or the Chi-square test. A significant statistical difference is one with a *p* value lower than 0.05.

## Results

### Clinical characteristics of patients

90 patients with UTUC who underwent LRNU, including 47 patients in the pre-ERAS group and 43 patients in the ERAS group, were included in this study. The findings of our initial analysis of the fundamental clinical features of the two groups (Table 2) revealed that there was no statistically significant difference between them in terms of gender, age, BMI, smoking and alcohol history, tumor location, or tumor side (*p* > 0.05). The tumor size and histological grade did not substantially differ between the two groups (*p* = 0.408 and *p* = 0.577, respectively). The pathological T-stage did not significantly differ between the two groups (*p* = 0.054), with 2 Ta, 10 T1, 3 T2, 25 T3, and 3 T4 cases in the ERAS group and 4 Ta, 4 T1, 12 T2, 22 T3, and 5 T4 cases in the pre-ERAS group.

**Table 2:** Baseline information of the two groups of patients

Variables	Pre-ERAS	ERAS	*P* value
Age (y)	68.4 ± 9.5	68.7 ± 9.3	0.902
Sex [n (%)]			0.904
Male	29 (61.7)	26 (60.5)	
Female	18 (38.3)	17 (39.5)	
BMI (kg/m2)	23.9 ± 3.0	23.4 ± 2.9	0.478
Smoking history [n (%)]	11 (23.4)	13 (30.2)	0.464
Alcohol history [n (%)]	7 (14.9)	4 (9.3)	0.419
Side [n (%)]			
Right	20 (42.6)	21 (48.8)	0.550
Left	27 (57.4)	22 (51.2)	
Tumor site [n (%)]			0.509
Renal pelvis	20 (42.6)	23 (53.5)	
Ureter	24 (51.1)	17 (39.5)	
Both	3 (6.4)	3 (7.0)	
Grade [n (%)]			0.577
Low	11 (23.4)	8 (18.6)	
High	36 (76.6)	35 (81.4)	
Tumor size (cm)	3.5 (2.0,4.0)	3.5 (2.2,4.5)	0.408
Pathologic T stage [n (%)]			0.054
Ta	4 (8.5)	2 (4.7)	
T1	4 (8.5)	10 (23.3)	
T2	12 (25.5)	3 (7.0)	
T3	22 (46.8)	25 (58.1)	
T4	5 (10.6)	3 (7.0)	

### Postoperative outcomes

Each patient in the two groups successfully underwent surgery and recovered well, and we then compared their postoperative outcomes (Table 3). Patients in the ERAS group had significantly shorter total and postoperative LOS when compared to the pre-ERAS group [total LOS: 15.0 (13.0–20.0) vs. 21.0 (16.0–26.0), *p* < 0.001; postoperative LOS: 8.0 (7.0–9.0) vs. 11.0 (9.0–13.0), *p* < 0.001]. In terms of hospitalization costs, there was a significant decline in the ERAS group [56896.40 (48324.30-67498.01) vs. 64249.83 (55574.36-81581.82), *p* = 0.010]. The time to remove the catheter did not differ substantially between the two groups (*p* = 0.271), however drainage tubes were removed earlier in the ERAS group than in the pre-ERAS group [5.0 (4.0–7.0) vs. 7.0 (6.0–8.0), *p* < 0.001]. Compared with the pre-ERAS group, the ERAS group had considerably less postoperative fever (*p* = 0.011), and no other early postoperative complications were statistically different.

**Table 3:** Comparisons of the outcomes between pre-ERAS and ERAS

Outcomes	Pre-ERAS	ERAS	*P* value
Total LOS (d)	21.0 (16.0–26.0)	15.0 (13.0–20.0)	< 0.001
Postoperative LOS (d)	11.0 (9.0–13.0)	8.0 (7.0–9.0)	< 0.001
Hospitalization costs (yuan)	64249.83 (55574.36-81581.82)	56896.40 (48324.30-67498.01)	0.010
Catheter removal (d)	8.0 (5.0–10.0)	7.0 (5.0–9.0)	0.271
Drainage tube removal (d)	7.0 (6.0–8.0)	5.0 (4.0–7.0)	< 0.001
Complications [n (%)]			
Urinary retention	1 (2.1)	1 (2.3)	1.000
Gastrointestinal discomfort	9 (19.1)	6 (14.0)	0.509
Postoperative hypotension	6 (12.8)	2 (4.7)	0.270
Delirium	1 (2.1)	1 (2.3)	1.000
Fever	11 (23.4)	2 (4.7)	0.011
Postoperative cardiovascular accidents	0	1 (2.3)	0.478

## Discussion

UTUC has a low incidence among urologic tumors and RNU is the gold standard for UTUC treatment [2]. Open radical nephroureterectomy with removal of the bladder cuff is the classic surgical approach. With the increasing development of laparoscopic technology, LRNU has been widely used in the treatment of UTUC. Recent studies have shown that LRNU can achieve the same tumor control as open surgery, but has the advantages of less trauma, less bleeding, and faster recovery [4, 15]. The surgical procedure, however, unavoidably results in bodily trauma, which impairs the patient’s postoperative recovery by causing tension, pain, and catheter-related discomfort. ERAS is a multidisciplinary and comprehensive treatment approach that optimizes perioperative treatment planning and reduces physical and psychological traumatic stress in patients through a series of interventions to accelerate patient recovery, shorten hospital stays and improve patient prognosis [16]. Many studies have shown that ERAS protocols yield significant results in radical cystectomy, laparoscopic radical nephrectomy and laparoscopic radical prostatectomy [17–19], but few studies have explored their impact on LRNU in patients with UTUC. Our center integrates LRNU with the ERAS concept to promote patient recovery and improve treatment through perioperative interventions, which has good clinical application value.

On the basis of our center’s actual situation and understanding of ERAS, our ERAS plan for patients undergoing LRNU includes the following key elements: comprehensive preoperative education, bowel-free preparation, restriction of intravenous fluids, postoperative analgesia, early resumption of oral feeding, and early activity [20, 21]. In the process of eras implementation, people’s adherence to traditional concepts became a major obstacle. In our center, the emphasis on preoperative patient education and the strengthening of multidisciplinary cooperative management guarantee better implementation of ERAS [22, 23]. With the same indications, the ERAS group’s postoperative hospital stay and drainage tube removal time were shorter than those in the pre-ERAS group, suggesting that patients in the ERAS group recovered faster. After 8 h without food, the catabolic pathway dominates, leading to increased glycogen breakdown, reduced glucose absorption by muscle, and insulin levels are normal. The anabolic pathway dominates when carbohydrate fluids are given two hours before surgery, replenishing glycogen, increasing muscle glucose absorption, rising insulin, and inhibiting proteolytic metabolism [24]. Unwanted physiological reactions may be amplified further by the indirect consequences of increased subjective hunger and thirst during the recovery time from surgery. Studies have shown that a 6-hour preoperative fast and a 2-hour water fast are both feasible and don’t increase the risk of anesthetic aspiration [25]. The natural barrier of the intestine can be damaged by preoperative mechanical bowel preparation, which can also change the water and electrolyte balance, alter the intestinal flora, and lengthen the time it takes for the intestine to heal after surgery. Early transoral feeding and activity can promote the recovery of bowel function, reduce the risk of pneumonia and thrombosis, and accelerate recovery [26]. Limiting fluid intake helps hasten the patient’s recovery of intestinal function by preventing issues such as intestinal edema that can be brought on by excessive fluid intake [27]. The results of this study revealed that the ERAS group had a lower incidence of postoperative fever than the control group did, which may be attributable to the improved perioperative management of the ERAS group, which included intraoperative insulation, better postoperative analgesia, and early activity to reduce postoperative stress and inflammatory response. Compared to the control group, the ERAS group had a shorter overall hospital stay, which could decrease hospitalization expenses and increase bed turnover. We discovered that the patients in the ERAS group had lower hospitalization costs than those in the control group, which decreased the financial burden on patients and had some positive economic and social effects. As a result, the use of ERAS among UTUC patients ensures medical quality and safety, increases medical effectiveness, and has practical value.

AirSeal^®^ is a three-lumen trocar insufflation system that creates a valve-less pressure curtain by continuous pressure flow and the system can improve visualization of the surgical field, enable continuous smoke sucking and reduce CO_2_ absorption and consumption [28]. Recent articles comparing the use of the AirSeal^®^ system vs. standard insufflator in laparoscopic urological procedure which emphasize how the use of the AirSeal^®^ system reduces operating times, rate of complications and perioperative blood loss [29, 30]. However, there are currently no studies of the AirSeal^®^ system for LRNU. The retroperitoneal space is smaller and less dilatable than it is with the transperitoneal approach. Therefore, a stable pneumoperitoneum is more important for better exposure of the surgical area and smooth operation. The AirSeal^®^ system ensures a stable pneumoperitoneum and continuous smoke evacuation during surgery, providing a useful tool for the LRNU. At our center, the AirSeal^®^ system is currently only used for robotic-assisted laparoscopic surgery. We will then focus on the effect of using the AirSeal^®^ system on LRNU and combine it with our ERAS experience, which we believe will produce better results.

The retroperitoneal route we used for LRNU allows more direct and rapid access to the corresponding organs and lesions, which is more advantageous for the exposure and treatment of renal arteries and veins, while avoiding damage to large abdominal vessels and organs. However, it has the problem of narrow space for retroperitoneal surgery and difficulty in dealing with the lower ureteral. The transperitoneal approach can provide a relatively larger space and clearer anatomical landmarks for surgery, making it easier for the surgeon to perform the procedure. However, transperitoneal surgery may have a higher risk of intra-abdominal organ injury compared to retroperitoneal surgery. There is also a concern that the transperitoneal approach is associated with worse disease progression [31]. Recently, laparoscopic radical nephroureterectomy with only three trocars with transperitoneal approach has been reported [32, 33]. Compared with the retroperitoneal approach, the technique enables complete LRNU without patient or port repositioning, is minimally invasive, has a shorter operation time and better postoperative pain control but requires a high degree of laparoscopic skill. Improved postoperative pain control allows earlier mobility out of bed and promotes recovery, and we believe that this surgical approach combined with our ERAS experience further promotes overall recovery from surgery.

Our study has several limitations. The first is the absence of intraoperative blood loss and surgical time. This is the loss of information due to the hospital system update. All surgeries were carried out by senior surgeons in our oncology treatment group with the same qualifications, so it is considered no difference between the two groups. Furthermore, because the data for this study came from a single site and it was retrospective rather than randomized, it is possible that the findings are biased and that future validation will require a prospective, multicenter randomized trial.

## Conclusion

In the realm of LRNU, ERAS protocols are safe and practical for minimizing the LOS while accelerating the rehabilitation of patients undergoing LRNU. This study offers insights for enhancing ERAS protocols for UTUC patients even further.

## Electronic supplementary material

Below is the link to the electronic supplementary material.

Supplementary Material 1

## Data Availability

The data used in this study can be obtained from our corresponding author.

## Abbreviations

ERAS: Enhanced recovery after surgery
UTUC: Upper tract urothelial carcinoma
LRNU: Laparoscopic radical nephroureterectomy
LOS: Length of hospital stay
POD: Postoperative day
